# β-Elemene Reverses the Resistance of p53-Deficient Colorectal Cancer Cells to 5-Fluorouracil by Inducing Pro-death Autophagy and Cyclin D3-Dependent Cycle Arrest

**DOI:** 10.3389/fbioe.2020.00378

**Published:** 2020-05-08

**Authors:** Ruonan Zhang, Ting Pan, Yu Xiang, Mingming Zhang, Jiao Feng, Shuiping Liu, Ting Duan, Peng Chen, Bingtao Zhai, Xiaying Chen, Wengang Wang, Bi Chen, Xuemeng Han, Liuxi Chen, Lili Yan, Ting Jin, Ying Liu, Guohua Li, Xingxing Huang, Wenzheng Zhang, Yitian Sun, Qiujie Li, Qin Zhang, Lvjia Zhuo, Tian Xie, Qibiao Wu, Xinbing Sui

**Affiliations:** ^1^Department of Medical Oncology, Holistic Integrative Pharmacy Institutes, The Affiliated Hospital of Hangzhou Normal University, College of Medicine, Hangzhou Normal University, Hangzhou, China; ^2^Key Laboratory of Elemene Class Anti-Cancer Chinese Medicine of Zhejiang Province and Engineering Laboratory of Development and Application of Traditional Chinese Medicine from Zhejiang Province, Hangzhou Normal University, Hangzhou, China; ^3^State Key Laboratory of Quality Research in Chinese Medicines, Faculty of Chinese Medicine, Macau University of Science and Technology, Macau, China; ^4^Department of Medical Oncology, Sir Run Run Shaw Hospital, Zhejiang University, Hangzhou, China

**Keywords:** colorectal cancer, p53, β-elemene, 5-fluorouracil, autophagy, cell cycle, drug resistance

## Abstract

**Objective:**

Colorectal cancer is a malignant tumor of the digestive system with high morbidity and mortality. 5-fluorouracil remains a widely used chemotherapeutic drug in the treatment of advanced colorectal cancer, but chemotherapy drugs are prone to develop drug resistance, p53 deletion or mutation is an important reason for the resistance of colorectal cancer cells to 5-fluorouracil. β-elemene has been proved to have the potential of reverse chemotherapy drug resistance, but the mechanism is unknown. This study aimed to investigate the effect of β-elemene to 5-fluorouracil in drug-resistant p53-deficient colorectal cancer cells HCT116p53^–/–^, and determine the possible molecular mechanism of β-elemene to reverse 5-fluorouracil resistance.

**Methods:**

The effect of β-elemene on HCT116p53^–/–^ cell activity was detected by Cell counting Kit-8. Cell proliferation was detected by monoclonal plate. The apoptosis was detected by flow cytometry and western blot. The autophagy was detected by western blot, immunofluorescence and transmission electron microscope. Determine the role of Cyclin-related protein Cyclin D3 in β-elemene reversing the resistance of HCT116p53^–/–^ to 5-fluorouracil was detected by overexpression of Cyclin D3. The effect of β-elemene on the tumorigenic ability of p53-deficient colorectal cancer cells was detected establishing HCT116p53^–/–^ all line xenograft model.

**Results:**

For p53 wildtype colorectal cancer cells, β-elemene could augment the sensitivity of 5-fluorouracil, for p53-deficient colorectal cancer cells, β-elemene significantly inhibited cell proliferation in a concentration-dependent manner, and reversed the resistance of HCT116p53^–/–^ to 5-fluorouracil by inducing pro-death autophagy and Cyclin D3-dependent cycle arrest.

**Conclusion:**

β-elemene enhances the sensitivity of p53 wild-type cells to 5-fluorouracil, β-elemene can reverse the resistance of HCT116p53^–/–^ to 5-fluorouracil by inducing pro-death autophagy and Cyclin D3-dependent cycle arrest in p53-deficient colorectal cancer, which will provide a new method for the treatment of p53 deletion colorectal cancer patients.

## Introduction

Cancer is one of the most serious public health problems in the world and has become a major killer of human health. According to the results of cancer data published in 2020 (Siegel et al., 2020), it is estimated that there will be 147,950 new cancer cases worldwide in 2020, and the incidence of colon cancer is 8.5%, ranking third, with 53,200 cancer deaths, and the death rate of colon cancer will be 9%, ranking third. Statistics of cancer data in China showed that (Chen W. et al., 2016) the incidence of colon cancer in men accounts for 10.98%, the incidence of women accounts for 7.92%, ranks third; male mortality accounts for 9.17%, female mortality accounts for 6.05%, ranks fifth.

Colorectal cancer is a malignant gastrointestinal tumor with very high morbidity and mortality. Due to the lack of early symptoms, most patients with colorectal cancer are advanced when diagnosed, and their prognosis is poor. And because the incidence of colorectal cancer is increasing year by year, it has brought tremendous mental and economic pressure to society. Current treatments include surgery, radiotherapy, chemotherapy, molecular targeted therapy, immunotherapy, and traditional Chinese medicine. Chemotherapy is a routine treatment for patients with advanced colorectal cancer. At present, the commonly used chemotherapy regimens in the clinic include FOLFIRI regimen for the combination therapy of aflibercept + fluorouracil, Leucovorin, and Irinotecan (Chau et al., 2020); mFOLFOX7 regimen combining Oxaliplatin, calcium folate and fluorouracil (Li et al., 2014); FOLFOX regimen combination with monoclonal antibodies (Raimondi et al., 2019). Fluorouracil drugs represented by 5-fluorouracil (5-Fu) are the first choice for the treatment of advanced colorectal cancer.

5-Fu plays an anti-tumor role by inhibiting thymidine adenylate synthase activity, preventing methylation of deoxythymidylate, and impairing DNA synthesis and repairing ability (Saif and Diasio, 2016). The efficacy of 5-Fu is usually limited by its resistance, and its resistance mechanisms include primary and secondary resistance. Increased transcription and translation levels of thymidine phosphorylase are the main causes of primary 5-Fu resistance. At present, there is no clear explanation of the molecular mechanism of secondary resistance to 5-Fu. The resistance mechanisms that have been reported in the literature mainly include the following: (1) The loss of thymidine kinase (TK) activity that catalyzes the metabolism of 5-Fu to an active effective product results in 5-Fu resistance (Sakamoto et al., 2015); (2) Dihydropyrimidine dehydrogenase (DPD) is mainly used to catalyze the catabolism of 5-Fu, and its enhanced activity causes 5-Fu to break down in large quantities, making it difficult to achieve effective drug concentrations for treating tumors (Fidai et al., 2018); (3) Lack of reduced folic acid substrate; (4) Mutations of tumor suppressor genes such as p53 (Dominijanni and Gmeiner, 2018); (5) Abnormal activation of signaling pathways such as Ras and Akt (Zong et al., 2019); (6) Related to DNA repair Mutation or deletion. The above molecular mechanism can make tumor cells resistant to 5-Fu and affect the therapeutic effect. Therefore, the enhancement of 5-Fu sensitivity is a research hotspot and needs to be further explored.

In recent years, there have been many therapeutic drugs for colorectal cancer. Fluorouracil drugs represented by 5-Fu are the cornerstone of colorectal cancer chemotherapy (Gajjar et al., 2018). During the treatment of recurrent tumors, stubborn tumor cells will produce chemotherapy. Drug resistance will greatly reduce the efficacy of chemotherapy drugs. Therefore, drug resistance has become a major problem that needs to be urgently addressed in clinical tumor treatment (Shamekhi et al., 2019), and how to reverse the resistance of 5-Fu has also become the focus of research.

It is reported that p53 as a tumor suppressor protein can control 5-Fu catabolism through the expression of the key rate-limiting enzyme of pyrimidine-degrading dihydropyrimidine dehydrogenase (DPYD). When p53 is mutated or deleted, 5-Fu catabolism is out of control to make cells resistant to drugs (Gokare et al., 2017). According to the distribution of p53 mutations in cancer worldwide^1^, it is known that the p53 mutation rate of colorectal cancer is 60%, ranking second in the p53 mutation rate of all cancers. Among all gene mutations in colorectal cancer, the p53 mutation rate is second only to allophycocyanin (APC). p53 can regulate a variety of physiological processes and inhibit tumor cell growth and reproduction (Gao et al., 2020), so it is of great significance to study the relationship between p53 mutations and colorectal cancer.

The mechanisms by which 5-Fu acts on p53 and participates in cellular processes are as follows: 5-Fu directly acts on p53: (1) p53 can inhibit the expression of IKB kinase (IKBK). IKBK participates in the intracellular immune response caused by cytokines, inhibits the phosphorylation of inhibitory protein (IκB), and inhibits the activation of NF-κB, thus inhibits cell survival; (2) p53 inhibits intracellular phosphatidylinositol kinase (PI3K) expression, or activates AMP-dependent protein kinase (AMPK), thereby inhibiting mammalian target of rapamycin (mTOR) expression and causing autophagy in tumor cells (Petroni et al., 2020); (3) p53 can promote cell apoptosis by activating members of the cysteine-containing aspartic proteolytic enzyme (caspase) family, B lymphoma-2 gene (Bcl-2), and other apoptosis-related proteins; (4) p53 can also directly acts on p21, inhibiting the expression of cycle-related proteins and causing cell cycle arrest (Sun et al., 2019). 5-Fu also can indirectly acts on p53:(1) 5-Fu indirectly acts on p53 by activating mitogen-activated protein kinase (MAPK), c-Jun amino terminal kinase (JNK), etc., causing apoptosis, autophagy, and cycle arrest; (2) 5-Fu causes DNA damage, p53 expression is affected by proteins related to DNA repair, causing apoptosis, autophagy and cycle arrest (Hafner et al., 2019).

Elemene (1-methyl-1-vinyl-2,4-diisopropenyl-cyclohexane) is a natural compound, and it has been found in more than 50 different plants, including warm turmeric, earthy wood, etc. Elemene has three isomers α, β, γ-elemene. β-elemene is the most active ingredient in the treatment of various cancers, accounting for 85% of the total (Yao et al., 2008; Wu et al., 2009; Sun et al., 2016).

Modern pharmacological studies have shown that β-elemene can inhibit many types of cancer by regulating multiple signaling pathways and targeting genes or proteins without causing serious side effects (Lee et al., 2012). β-elemene used to be treated with variety of cancers, including lung cancer (Li et al., 2011), esophageal cancer (Chen X. et al., 2016), breast cancer (Guan et al., 2014), gastric cancer (Imanishi et al., 2019), liver cancer (Zhai et al., 2018), melanoma cancer (Shi et al., 2015), ovarian cancer (Li et al., 2013b), can also cross the blood-brain barrier to treat gliomas (Feng et al., 2017). Its main antitumor mechanisms include inhibition of tumor cell proliferation, elimination of tumor cells, induction of apoptosis and differentiation, induction of autophagy, induction of cell cycle arrest, inhibition of tumor metastasis and immune regulation (Chen et al., 2017; Satyavarapu et al., 2018; Zhang et al., 2018).

In vitro research and clinical practice also proved that β-elemene can reverse the resistance of tumor cells to a variety of anticancer drugs (Mu et al., 2016) including chemotherapeutics, tyrosine kinase inhibitor gefitinib, etc. However, the target of β-elemene and the effect of reversing the resistance to 5-Fu in p53-deficient colorectal cancer in clinical treatment have not been reported.

As many tumors develop resistance to conventional chemotherapy and radiotherapy soon after treatment, even at the beginning of treatment, this has become one of the main reasons for the failure of tumor treatment. The strategy of western medicine in overcoming tumor resistance is to replace with new antitumor drugs. As a result, new resistance or even multidrug resistance is easy to be produced, and Chinese medicine has incomparable advantages in overcoming antitumor resistance. Therefore, studying the role of β-elemene in reversing 5-Fu resistance and clarifying its target and molecular mechanism has particularly important clinical significance, which will provide important theoretical basis and treatment strategies for the treatment of p53-deficient colorectal cancer patients.

## Materials and Methods

### Cell Lines and Culture Methods

The human colorectal cancer lines HCT116p53^+/+^ and HCT116p53^–/–^ were a gift from Mr. Liu Jiang, Hangzhou Normal University. The cells were cultured in McCoy’s 5A medium (GENOM, GNM16600, China), supplemented with 10% heat-inactivated fetal bovine serum at 37°C with 5% CO_2_ and 95% humidity, and the culture medium was usually changed every 2 days. The fetal bovine serum was purchased from Corille (184590, Australia).

### Antibodies and Chemicals

Anti-GAPDH (#5174) antibody and p21 (#2947), p27 (#3686) were purchased from Cell Signaling Technology (CST). Anti-p53 (sc-126) antibody was obtained from Santa Cruz. Autophagy marker LC3B (#3868) and Beclin-1 (#3738) was purchased from CST. Apoptosis marker Active caspase-3 (BS7004) was obtained from Bioworld, Cleaved PARP (#5625) was purchased from CST. Cell cycle protein CDK2 (#2546), CDK4 (#12790), CDK6 (#3136), Cyclin D3 (#2936) were purchased from CST. Anti-rabbit IgG, HRP-linked Antibody (#7074) and Anti-mouse IgG, HRP-linked Antibody (#7076) were also obtained from CST.

β-elemene was obtained from LKT lab (E4418, purity ≥98%), β-elemene was dissolved in ethanol (02483, Supelco). 5-Fluorouracil (HY-90006), Bafilomycin A1 (HY-100558) and Hydroxychloroquine (HCQ) (HY-B1370) were purchased from MCE. Ampicillin (A8180) was obtained from Solarbio. Kanamycin (K8020) was obtained from Solarbio. *Trans*5α Chemically Competent Cell (CD201-01) was purchased from Transgene. LB Broth (L1010) was obtained from Solarbio. LB agar (L1015) was obtained from Solarbio. AxyPrep Plasmid Miniprep Kit (AP-MN-P-250G) was purchased from Axygen. pCMV6-Entry Tagged Cloning Vector (PS100001) and Cyclin D3 plasmid (RC227101) were obtained from Origene. pMRX-IP-GFP-LC3-RFP-LC3ΔG (#84572) was purchased from addgene. Lipofectamine^TM^ 2000 Transfection Reagent (11668019) and ProLong^®^ Gold Antifade Reagent (P10144) were purchased from Thermo Fisher Scientific. DAPI (C0065) was purchased from Solarbio.

### Cell Proliferation and Viability Assays

Cell viability was evaluated using the Cell Counting Kit-8 (CCK-8) (LJ621, Dojindo, Japan) according to the manufacturer’s instructions. HCT116p53^+/+^ and HCT116p53^–/–^ cells were seeded in 96-well flat bottom microtiter plates at a density of 5,000 cells per well. Incubated overnight, add 100 μl of media containing different concentrations of β-elemene, and incubate in the incubator for 24, 48, and 72 h. After incubation, aspirate the medium from the 96-well plate and add the medium mixed with CCK8 (medium: CCK8 = 10: 1) Continue to incubate for 4 h at 37°C. After 4 h, stop the culture. The optical density values were determined at least in triplicate against a reagent blank at a reference wavelength of 450 nm using a spectrophotometer microplate reader. The experiment was repeated three times.

### Clone Formation Assays

HCT116p53^+/+^ and HCT116p53^–/–^ cells were seeded in 10 cm dish at a density of 1,000 cells per well, and then culture them in a cell incubator at 37°C, 5% CO_2_ and saturated humidity. When the clone can be seen under the microscope, add the medium containing different drugs (control, 5-Fu, β-elemene, 5-Fu + β-elemene), change the new culture medium once every 3 days, and continue to culture for 2 weeks. When visible macroscopic clones appear, the culture is terminated. The supernatant was discarded and carefully washed twice with PBS. Fix with 4% formaldehyde for 15 min. Then remove the fixing solution, add an appropriate amount of 0.25% crystal violet staining solution, dye for 15–20 min, then slowly wash away the staining solution with running water, and air dry. Invert the plate and overlay a grid of transparencies and count the clones directly with the naked eye, or count more than 50 cells with a microscope (low magnification). The experiment was repeated three times.

### Cell Apoptosis Assays

Flow cytometry was used to detect apoptosis induced by 5-Fu, β-elemene, and 5-Fu + β-elemene. Using the Annexin V-FITC/PI Apoptosis Detection Kit (556547) from BD in the United States, each operation step is strictly performed according to the instructions. HCT116p53^+/+^ and HCT116p53^–/–^ cells were seeded in 6 cm dish at a density of 5 × 10^5^ cells per well, and then culture them in a cell incubator at 37°C, 5% CO_2_ and saturated humidity. Incubated overnight, add different treatment group media (control, 5-Fu, β-elemene, 5-Fu + β-elemene) for 24 h, collect all cell culture fluids and cells, centrifuge at 800 *g* for 5 min and remove the supernatant. Wash the cells with cold PBS, centrifuge, discard the supernatant, then resuspend the cells by adding 1 ml of 1 × binding buffer, and adjust the cell concentration to 10^6^ cells/ml. Add 100 μl (10^5^ cells) of cell suspension to the flow tube, add 5 μl FITC-Annexin V and 5 μl PI to each flow tube. Mix the cells with the staining agent, and leave it in the dark for 15 min at room temperature. Then add 400 μl of 1 × binding buffer to each flow tube, and test it on the machine. Annexin V-FITC shows green fluorescence and PI shows red fluorescence. The experiment was repeated three times.

### Cell Transfection

The Lipofectamine^TM^ 2000 Transfection Reagent (11668019) was used to transfect the HCT116 p53^–/–^ cells. Transfection was performed according to the manufacturer’s instructions. HCT116 p53^–/–^ cells were seeded in 6 cm dish at a density of 5 × 10^5^ cells per well. Incubated overnight, the cell fusion degree reached 70–80%. Add 50 μl OPTI-MEM to two 1.5 ml EP tubes, add 3 μg plasmid to one tube, 9 μl Lipofectamine 2000 to one tube, and add OPTI-MEM containing Lipofectamine 2000 to OPTI-MEM with plasmid. After mixing, leave it at room temperature for 5 min, then add it dropwise to the culture well and shake gently, mix it in the incubator and incubate for 6 h, then change to complete medium and continue to culture.

### Western Blot

HCT116p53^+/+^ and HCT116p53^–/–^ cells were seeded in 6 cm dish at a density of 6 × 10^5^ cells per well. Incubated overnight, add different treatment group media (control, 5-Fu, β-elemene, 5-Fu + β-elemene) for 24 h. Cells were harvested and lysed using the RIPA buffer (P0013B, Beyotime) in the presence of a phenylmethyl sulfonylfluoride (PMSF) (#8553, CST). Protein concentration was determined using the BCA Protein Assay Kit (P0009, Beyotime). Equivalent amounts of protein were resolved and mixed with 5× SDS-PAGE protein sample buffer (P0015, Beyotime), electrophoresed in SDS-PAGE, transferred to PVDF membranes (Merck Millipore, Billerica, MA, United States). The blotted membranes were blocked with 5% skim milk for 1 h and incubated with primary antibodies overnight at 4°C. Day 2, washed with TBST (CW0043S, CWBIO), then incubated with suitable HRP-conjugated second antibodies and subjected to enhanced chemiluminescent staining using an ECL detection system (Bio-Rad). All experiments were conducted in triplicate.

### Immunofluorescence Assay

For immunofluorescence assays, 3 × 10^5^ cells were seeded into 6-well plates with coverslips, transiently transfected the plasmid with RFP-GFP-LC3B into HCT116p53^–/–^ cells for 48 h, and treated with control, 5-Fu, β-elemene, and 5-Fu + β-elemene for 24 h. Then the cells were fixed in 4% paraformaldehyde, washed with PBS and stained with 0.05% DAPI for 15 min. Finally, washed with PBS and mounted with anti-fluorescent quencher (ProLong^®^ Gold Antifade Reagent). Images were obtained with the laser scanning confocal microscope (Nikon, Japan).

### Transmission Electron Microscopy

HCT116p53^+/+^ and HCT116p53^–/–^ cells were seeded in 6 cm dish at a density of 6 × 10^5^ cells per well. Incubated overnight, add different treatment group media (control, 5-Fu, β-elemene, 5-Fu + β-elemene, Bafilomycin A1, 5-Fu + β-elemene+ Bafilomycin A1) for 24 h. Collected cells and fixed with 2.5% glutaraldehyde, then dehydrated, embedded, sectioned, stained, and finally observed for autophagic vacuole using a Transmission electron microscopy (TEM).

### Animal Experiments

Eighteen female nude mice of 6-week-old BALB/c thymus deletion weighing about 20 g were purchased. Nude mice were randomly divided into six groups: control group, 5-Fu group, β-elemene group, HCQ group, 5-Fu + β-elemene group, and 5-Fu + β-elemene + HCQ group. 10^6^/100 μl of the cell suspension was inoculated under the skin of the nude mice. On the third day of vaccination, the nude mice in the control group were injected intraperitoneally with PBS daily. The 5-Fu group was intraperitoneally injected with 20 mg/kg/2d 5-Fu. The β-elemene group was intraperitoneally injected with 100 mg/kg/d β-elemene. The HCQ group intraperitoneally injected 60 mg/kg/d HCQ. The 5-Fu + β-elemene group was intraperitoneally injected with 20 mg/kg/2d 5-Fu and 100 mg/kg/d β-elemene. HCQ group intraperitoneally injected 60 mg/kg/d HCQ. 5-Fu + β-elemene + HCQ group intraperitoneally injected 20 mg/kg/2d 5-Fu and 100 mg/kg/d β-elemene and 60 mg/kg/d HCQ. Record daily, and measure the long and short diameters of the tumor every 3 days. Tumor volume = (shortest diameter)^2^ × longest diameter × 0.5. On the 24th day after vaccination, all nude mice were sacrificed by cervical dislocation, with time as the abscissa and tumor volume as the ordinate to make growth curves.

### Statistical Analysis

Data are expressed as means ± SD of three independent experiments. Statistical analysis was performed using Prism 7.0 GraphPad Software. The significance of differences between groups was determined using t-test. A *p*-value <0.05 was considered statistically significant.

## Results

### 5-Fu and β-Elemene Inhibit Proliferation of Colorectal Cancer Cells

**FIGURE 1:**
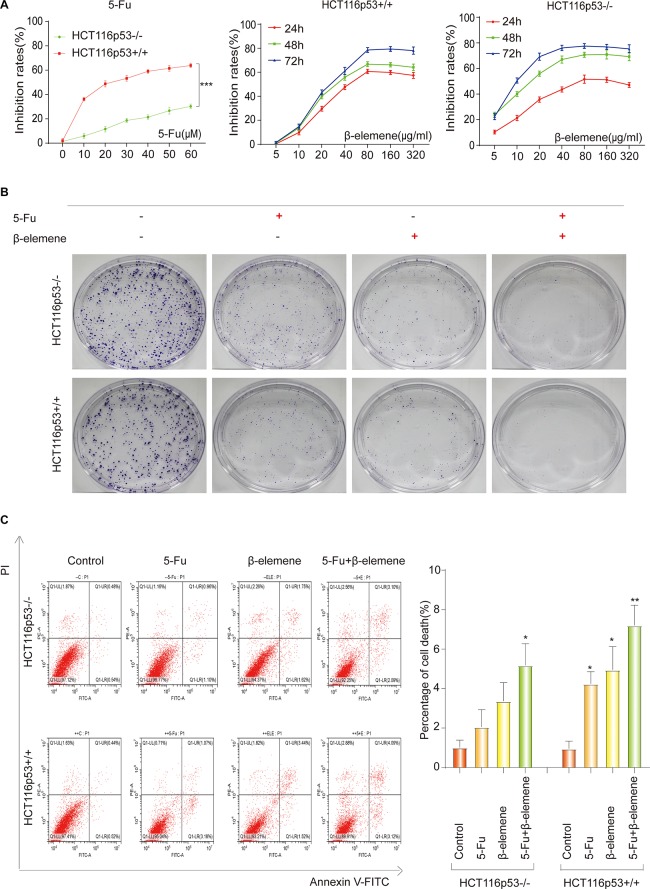
5-Fu and β-elemene inhibits proliferation of colorectal cancer cells. **(A)** HCT116p53 ^+/+^ and HCT116p53^–/–^ cells were treated with different concentrations of 5-Fu for 24 h, or treated with β-elemene for 24, 48, and 72 h, the cell viability was examined by CCK8; **(B)** the cell proliferation was detected by a clone formation assays; **(C)** the apoptosis rate of cells was examined by flow cytometry. (^∗^*p* < 0.05, ^∗∗^*p* < 0.01, *n* = 3).

Previous literature has confirmed that p53-deficient colorectal cancer cells HCT116p53^–/–^ are resistant to 5-Fu (Sui et al., 2014). So we investigated the sensitivity of 5-Fu in HCT116p53^+/+^ and HCT116p53^–/–^ cells. The two cells were treated with various concentrations of 5-Fu for 24 h and the cell viability was assessed by the CCK-8. The results showed that 5-Fu inhibits cell proliferation in HCT116p53^+/+^ cells in a dose-dependent manner. HCT116p53^+/+^ cells were highly sensitive to 5-Fu, but very few dying cells emerged in HCT116p53^–/–^ cells after 5-Fu treatment (^∗∗∗^*p* < 0.001), indicating that HCT116p53^–/–^ cells may be insensitive or resistant to 5-Fu (Figure 1A). The 24 h IC_50_ of 5-Fu for HCT116p53^+/+^ cells was 23.39 μM, so we used 20 μM 5-Fu in HCT116p53^+/+^ cells and HCT116p53^–/–^ cells for 24 h in subsequent experiments.

To study the effect of β-elemene on the proliferation of HCT116p53^+/+^ and HCT116p53^–/–^ cells, the cells were treated with various concentrations of β-elemene for 24, 48, and 72 h, the cell inhibited rates were assessed by the CCK-8 (Figure 1A). The results showed that β-elemene inhibit cell proliferation in a dose-dependent manner. As the concentration of β-elemene increased, the inhibition rates of HCT116p53^+/+^ cells was increased. The IC_50_ of 24, 48, and 72 h were 42.20, 39.17, and 38.27 μg/ml, respectively. HCT116p53^–/–^ cells also had a concentration and time dependence on β-elemene. As the concentration and time of β-elemene increased, the inhibition rates on HCT116p53^–/–^ cells was increased. The IC_50_ of the HCT116p53^–/–^ cells at 24, 48, and 72 h were 71.75, 14.88, and 9.40 μg/ml, respectively. Consequently, we used 40 μg/ml β-elemene in HCT116p53^+/+^ cells and HCT116p53^–/–^ cells for 24 h in subsequent experiments.

### Combination of 5-Fu and β-Elemene Can Increase the Sensitivity of HCT116p53^+/+^ Cells to 5-Fu and Reverse the Resistance of HCT116p53^–/–^ Cells to 5-Fu

To investigate the effect of β-elemene on the sensitivity of 5-Fu to HCT116p53^–/–^ cells and HCT116p53^+/+^ cells, we performed a clone formation assays to detect cell proliferation. Every cell line divided into four groups: control group, 5-Fu group, β-elemene group, 5-Fu + β-elemene group (Figure 1B). The results showed that both 5-Fu and β-elemene can inhibit the proliferation of HCT116p53^+/+^ cells, while the inhibition effect of 5-Fu + β-elemene is more obvious. This may indicate that the sensitivity of 5-Fu is enhanced by β-elemene. For HCT116p53^–/–^ cells, 5-Fu has little effect on the proliferation of cells, but β-elemene can inhibit clone formation of HCT116p53^–/–^ cells. The combination of 5-Fu and β-elemene can significantly inhibit the reproduction of HCT116p53^–/–^ and reduce the colony formation rates. This may indicate that β-elemene reversed 5-Fu resistance.

To further investigate whether β-elemene can enhance the sensitivity of 5-Fu to HCT116p53^–/–^ cells and reverse the resistance of 5-Fu to p53-deficient colorectal cancer cells, we used flow cytometry to detect the apoptosis rates of cells (Figure 1C). The results showed that HCT116p53^–/–^ cells were not sensitive to 5-Fu, and cells have little apoptosis in the 5-Fu group. More cells apoptosis in β-elemene group, but it was not obvious compared with the control group. A large number of cells died in the 5-Fu + β-elemene group, and apoptosis was significantly higher than control group (^∗^*p* < 0.05), indicating that β-elemene can reverse the resistance of 5-Fu to p53-deficient colorectal cancer cells. For HCT116p53^+/+^ cells, both 5-Fu and β-elemene can cause apoptosis (^∗^*p* < 0.05). And the apoptosis of the 5-Fu + β-elemene group was significantly higher than the control group (^∗∗^*p* < 0.01), indicating that β-elemene can increase the sensitivity of 5-Fu.

### β-Elemene Reverses the Resistance of HCT116p53^–/–^ Cells to 5-Fu by Inducing Pro-death Autophagy

**FIGURE 2:**
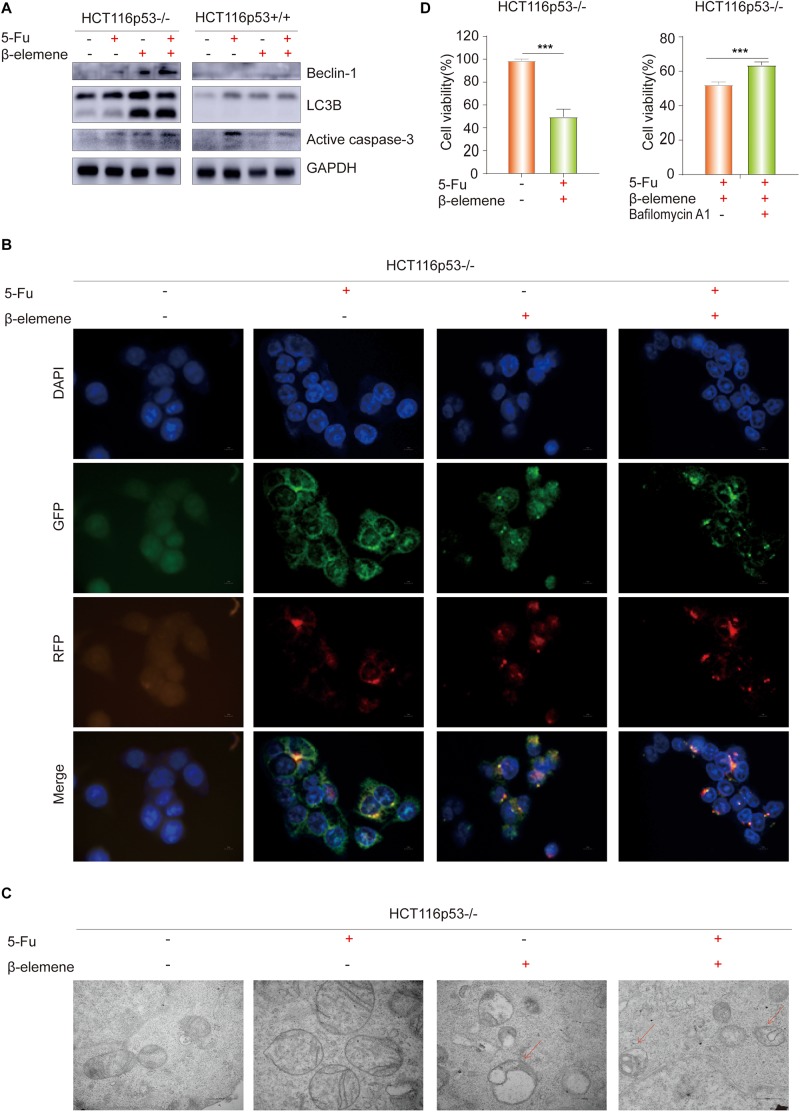
β-elemene reverse the resistance of HCT116p53^–/–^ cells to 5-Fu by inducing pro-death autophagy. **(A)** HCT116p53^+/+^ and HCT116p53^–/–^ cells were treated with control, 5-Fu, β-elemene, 5-Fu + β-elemene, the protein expression of LC3B, Beclin-1, and Active caspase-3 were examined by Western blot; **(B)** HCT116p53^–/–^ cells were treated with control, 5-Fu, β-elemene, 5-Fu + β-elemene, the autophagy flow was observed by immunofluorescence, red arrow indicates autolysosomes; **(C)** HCT116p53^–/–^ cells were treated with control, 5-Fu, β-elemene, 5-Fu + β-elemene, the occurrence of autophagy was observed by transmission electron microscope; **(D)** HCT116p53^–/–^ cells were treated with 5-Fu + β-elemene, 5-Fu + β-elemene + Bafilomycin A1, the cell viability was examined by CCK8. (***p < 0.001, *n* = 3).

To determine how β-elemene reverse the resistance of HCT116p53^–/–^ cells to 5-Fu, the HCT116p53^+/+^ cells and HCT116p53^–/–^ cells were treated with 5-Fu, β-elemene, and 5-Fu + β-elemene for 24 h, and then the protein was extracted for Western blot. The results showed that LC3B, an autophagy marker protein, was significantly increased in β-elemene group and 5-Fu + β-elemene group in HCT116p53^–/–^ cells (Figure 2A). Besides, the autophagy protein Beclin-1 showed the same result. However, there was no change in autophagy protein in HCT116p53^+/+^ cells, indicating that there was no autophagy in HCT116p53^+/+^ cells. In HCT116p53^–/–^ cells, Active caspase-3, an apoptosis protein, was increased in β-elemene group and 5-Fu + β-elemene group, indicating that β-elemene increased apoptosis by promoting autophagy of HCT116p53^–/–^ cells.

LC3B is a hallmark protein of autophagy. Through immunofluorescence-labeled LC3B, the localization of LC3B in cells can be observed under a fluorescence microscope. The amount of LC3BII can reflect the autophagy activity of cells, the number of autophagy particles indicates the degree of autophagy (Kaizuka et al., 2016). Increased autophagy caused by β-elemene was further confirmed by immunofluorescence (IF) staining, we transiently transfected the plasmid with RFP-GFP-LC3B into HCT116p53^–/–^ cells, and treated with 5-Fu, β-elemene, and 5-Fu + β-elemene. The IF staining data revealed that there were no autophagy particles in the control group and 5-Fu group, autophagy particles were increased in the β-elemene group and 5-Fu + β-elemene group, and red fluorescence enhancement in the 5-Fu +β-elemene group, which proved that β-elemene promoted autophagy in HCT116p53^–/–^ (Figure 2B).

Observing the formation and number of autophagosomes with a transmission electron microscope (TEM) is the most commonly used method for measuring autophagy (Levy et al., 2017). Phagocytic vesicles are characterized by a crescent or goblet-shaped, double-layered or multi-layered membrane with a tendency to surround cytoplasmic components. Autophagosomes is characterized by a vacuole-like structure with double or multilayer membranes, which contain cytoplasmic components such as mitochondria, endoplasmic reticulum, and ribosomes. Autophagosome are characterized by a monolayer membrane and cytoplasmic components that have been degraded (Amaravadi et al., 2019). To better observe the occurrence of autophagy, TEM was performed after the cells were treated with control, 5-Fu, β-elemene, 5-Fu + β-elemene (Figure 2C). It was observed that the mitochondrial bilayer membrane structure in the control group and 5-Fu group was intact, indicating that there was no autophagy. The mitochondrial structure in the β-elemene group and 5-Fu + β-elemene group was destroyed. Red arrow indicates the fusion of autophagosomes and lysosomes. The number of Autophagolysosomes increased in the 5-Fu + β-elemene group, indicating that β-elemene can induce autophagy in HCT116p53^–/–^ cells and increase the formation of autophagosome.

To verify the type of autophagy induced by β-elemene in HCT116p53^–/–^ cells, we performed a CCK-8 assay on HCT116p53^–/–^ cells (Figure 2D). The results showed that 5-Fu + β-elemene can significantly reduced the activity of HCT116p53^–/–^ cells (^∗∗∗^*p* < 0.001). Then we added autophagy inhibitor Bafilomycin A1 (20 nM) to HCT116p53^–/–^ cells, compared with the 5-Fu + β-elemene group, the cell activity of 5-Fu + β-elemene+ Bafilomycin A1 group was significantly increased (^∗∗∗^*p* < 0.001), indicating that β-elemene induced pro-death autophagy in HCT116p53^–/–^ cells.

### β-Elemene Reverses the Resistance of HCT116p53^–/–^ Cells to 5-Fu by Inducing Cyclin D3-Dependent Cycle Arrest

**FIGURE 3:**
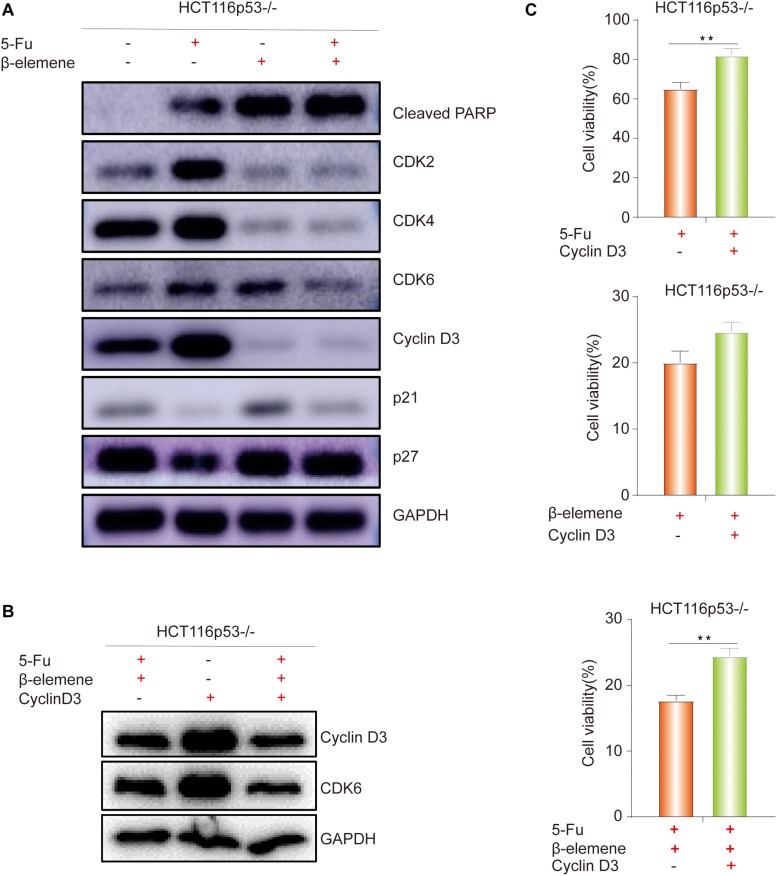
β-elemene reverse the resistance of HCT116p53^–/–^ cells to 5-Fu by inducing Cyclin D3- dependent cycle arrest. **(A)** HCT116p53^–/–^ cells were treated with control, 5-Fu, β-elemene, 5-Fu + β-elemene, the expression of cell cycle-related proteins were examined by Western blot; **(B)** HCT116p53^–/–^ cells were treated with 5-Fu + β-elemene, Cyclin-D3, 5-Fu + β-elemene + Cyclin D3, the protein expression of Cyclin D3 and CDK6 were examined by Western blot; **(C)** Overexpression Cyclin D3 of HCT116p53^–/–^ cells and treated with 5-Fu, β-elemene, 5-Fu + β-elemene, the cell viability was examined by CCK8. (**p < 0.01, *n* = 3).

As everyone knows that p53 directly activates p21, which can inhibit the cell cycle-related protein such as Cyclin D and CDK4/6, leading to G1 phase cell arrest (Cayrol et al., 1998). Cyclin D3 can directly combine with CDK4/CDK6 to form a complex and promote the cell cycle process. Therefore, we speculated that β-elemene can inhibit cell growth by inhibiting the cell cycle process. To verify this view, Western blot was used to detect cell cycle-related proteins. The results showed that the expression of Cleaved PARP protein was increased in β-elemene group and 5-Fu + β-elemene group, indicating that β-elemene increased the apoptosis of HCT116p53^–/–^ cells and reverse 5-Fu resistance. The expression of CDK2/4/6 and Cyclin D3, which are related to G1 phase proteins, were decreased in β-elemene group and 5-Fu + β-elemene group, indicating that β-elemene can inhibit the expression of G1 phase proteins and cause cycle arrest (Figure 3A).

### Effect of Overexpression of Cyclin D3 on β-Elemene Reverses the Resistance of HCT116p53^–/–^ Cells to 5-Fu

The above results showed that β-elemene induced cell cycle arrest. In the β-elemene group and 5-Fu + β-elemene group, the expression of the cell cycle-related protein Cyclin D3 was significantly reduced than the control group. Because Cyclin D forms a complex with CDK4/6, which can lead to G1 phase arrest. Therefore, we supposed that the cell cycle arrest caused by β-elemene is related to Cyclin D3. To verify this guess, we transiently transfected the Cyclin D3 plasmid and the empty plasmid into HCT116p53^–/–^ cells, respectively. After transfection, they were divided into control group and 5-Fu + β-elemene group. Western blot results showed that overexpressing Cyclin D3 increased the expression of Cyclin D3 protein and CDK6 protein, while the expression of Cyclin D3 protein and CDK6 protein were reduced by treated with 5-Fu + β-elemene (Figure 3B). CCK8 results showed that compared with the transfection of empty plasmid, overexpression of Cyclin D3 can increase the sensitivity of 5-Fu (^∗∗^*p* < 0.01), and also increased the activity of cells in β-elemene group, but it was not obvious. Overexpression of Cyclin D3 can also significantly increased the activity of cells in the 5-Fu + β-elemene group (^∗∗^*p* < 0.01), indicating that β-elemene can reverse the resistance of HCT116p53^–/–^ cells to 5-Fu by inhibiting the expression of Cyclin D3 (Figure 3C).

### Effect of β-Elemene Combined With 5-Fu on Tumorigenicity of HCT116p53^–/–^ Cells *in vivo*

**FIGURE 4:**
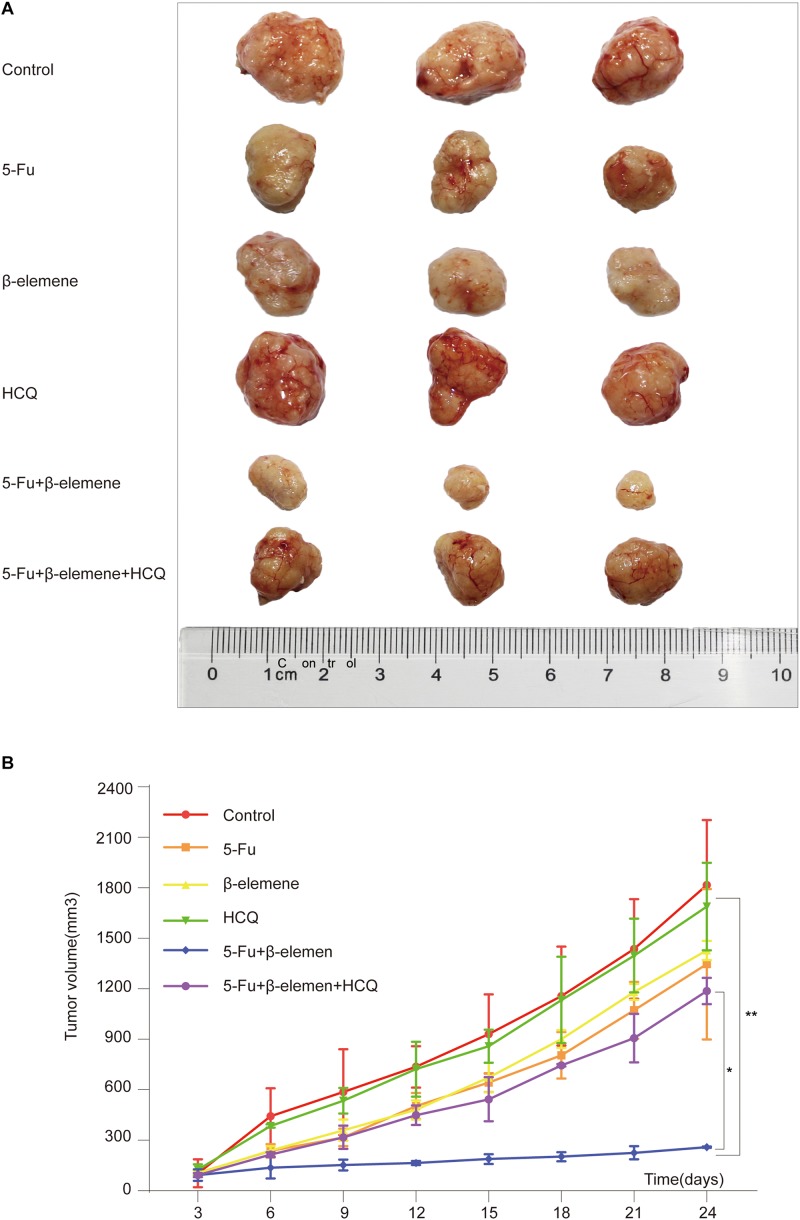
Effect of β-elemene combined with 5-Fu on tumorigenicity of HCT116p53^–/–^ cells in vivo. **(A)** The tumor tissue; **(B)** Tumor volume curve. (*p < 0.05, **p < 0.01, *n* = 3).

To investigate the effect of 5-Fu + β-elemene on tumorigenicity of tumor cells in vivo, we established HCT116p53^–/–^ all line xenograft model, which was randomly divided into six groups: control group, 5-Fu group, β-elemene group, HCQ group, 5-Fu + β-elemene group, and 5-Fu + β-elemene + HCQ group. At the end of the experiment, the tumor tissue was taken and photographed. The results showed that the tumor in the control group was completely uncontrolled and grew wildly. In the HCQ group, there has no effect on tumor growth. In the 5-Fu group and β-elemene group, the tumor volume was suppressed, while the tumor volume suppression rates was lower. In the 5-Fu + β-elemene group, tumor volume was significantly inhibited (^∗∗^*p* < 0.01), and the effect was higher than β-elemene alone, but HCQ could alleviate this effect (^∗^*p* < 0.05) (Figures 4A,B).

## Discussion

Autophagy is the process of lysosomal degradation. In the process, the living body can remove intracellular waste and rebuild the structure to promote cell growth and development, maintain the stability of the intracellular environment and the balance of protein metabolism (Ramkumar et al., 2017). The autophagy process is divided into five different phases: the initial phase, the vesicle nucleation phase, the vesicle extension phase, the vesicle fusion phase, and the degradation products within the capsule (Kondo et al., 2005). Autophagy is tightly regulated by many intracellular signaling pathways and molecules. It acts as a regulator of pathogenesis and is a potential therapeutic target in a variety of diseases by regulating apoptosis, inflammation, pathogen clearance, immune response, and other cellular processes (Kondratskyi et al., 2018). The induction of autophagy plays a pro-death or pro-survival role in the treatment of cancer by chemotherapeutic drugs, which is related to the therapeutic effect of anticancer drugs and chemotherapeutic resistance. Autophagy serves as a drug resistance mechanism against chemotherapeutic drug therapy. And autophagy-mediated cell death mechanism is beneficial to the efficacy of anticancer drugs and improve the sensitivity of chemotherapeutic drugs including 5-Fu (Sui et al., 2013). In this paper, β-elemene reverses the resistance of p53-deficient colorectal cancer cells to 5-Fu by inducing pro-death autophagy.

So far, based on the delivery of cytosolic substances to lysosomes and substrates, three different main autophagy mechanisms have been distinguished: microautophagy, macroautophagy, and chaperonemediated autophagy. Accompanying protein-mediated autophagy and microautophagy involve direct unfolded soluble proteins into the lysosome as well as nonspecific phagocytosis of the cytoplasm, including organelles. Macroautophagy is a necessary, conservative autophagy process that degrades intracellular components, including soluble proteins, aggregates, organelles, polymer complexes, and foreign bodies (Ravegnini et al., 2017).

Generally, cell autophagy will maintain the stability of the intracellular microenvironment since it is maintained at a lower level. The lower level of autophagy plays a role in protecting cells, mainly degrading some large molecular proteins or organelles damaged by aging. Autophagy can inhibit the formation and growth of tumor cells (Yang and Klionsky, 2010; Sui et al., 2011). However, when the cell is subjected to certain external stimuli, such as starvation, drugs, hypoxia, etc., the level of autophagy that occurs in the cell will greatly increase, related autophagy proteins will be activated, and autophagy-related pathways will be activated. After the formation of autophagosomes, membrane phospholipids and intracellular proteins begin to be degraded by lysosomes. At the same time, autophagy can provide energy for cell survival through the lysosome degradation of organelles (Xin et al., 2017). However, excessive or sustained autophagy can also cause cytotoxicity, promoting cell death rather than survival.

Although the occurrence of autophagy is very common and the detection methods are diverse, the simplest is to detect LC3B, which is an autophagy marker protein (Janku et al., 2011). It can be processed from precursor LC3B into cytosolic soluble LC3B?, while exposing glycine residues are activated by Atg7. It is modified into LC3B?, which can bind to the membrane and is used as a marker of autophagosomes to locate in pre- and autophagosomes. The higher the content, the greater the degree of autophagy. Transient exogenous GFP-RFP-LC3B in this experiment can only prove the autophagosomes of cells, and this method is a benchmark for detecting cell autophagy (Nuta et al., 2019). In this experiment, we observed a large number of autophagosomes in HCT116p53^–/–^ cells treated with β-elemene, which act as an autophagic marker indicating the occurrence of autophagy. There are two types of autophagy induced by chemotherapeutic drugs: one is protective autophagy, as a mechanism to maintain the stability of the intracellular environment, and the other is the cause of drug resistance, which is pro-death autophagy. Autophagy as a death mechanism promotes cell death (Hu et al., 2012; Zou et al., 2012).

β-elemene, as a new plant-derived compound, has a broad anti-cancer spectrum and may be further developed into anti-metastatic drugs. There are many reports on its inhibitory effect on tumors and its role in reversing drug resistance, but its underlying molecular mechanism is still unclear. In our study, the changes in cell autophagy particles were observed after transient transfection GFP-RFP-LC3B plasmid. Autophagy particles were increased in the β-elemene group and 5-Fu + β-elemene group, and Western blot results showed that the expression of LC3B? significantly increased after β-elemene treatment, indicating that β-elemene can increase the apoptosis of p53-deficient colorectal cancer cells by inducing autophagy, thereby increasing the sensitivity of 5-Fu. Besides, by measuring the cell viability after adding the autophagy inhibitor Bafilomycin A1, the cell viability was increased after the inhibition of autophagy, indicating that β-elemene induces pro-death autophagy in HCT116p53^–/–^ cells and reverse the resistance of HCT116p53^–/–^ cells to 5-Fu.

Previous studies have shown that β-elemene can achieve anti-tumor effects by inhibiting the cell cycle (Li et al., 2013a). In this experiment, cell cycle-related proteins were detected, the results showed that the expression of CDK2, CDK4, CDK6, and Cyclin D3 proteins were reduced in β-elemene group and 5-Fu + β-elemene group, indicating that the cell cycle process is blocked by β-elemene and the cell proliferation ability is reduced. Overexpression the Cyclin D3 enhances the activity of HCT116p53^–/–^ cells induced by 5-Fu + β-elemene, indicating that β-elemene can inhibit the expression of cell cycle-related proteins and cause Cyclin D3-dependent cell cycle arrest in HCT116p53^–/–^ cells.

The core part of the cycle mechanism is composed of D-type cyclins Cyclin D1, Cyclin D2, and Cyclin D3. Cyclin-dependent proteins are activated by cyclins and can form complexes, which can regulate the cell cycle from G1 to S change. Tumor cells have stronger proliferation ability than normal cells, so most tumor cells have more cells in the S phase, and Cyclin D protein expression is higher. If Cyclin D-CDK4/CDK6 complex expression is inhibited, it can inhibit the cell cycle process and promote cell aging, which is expected to become a new therapeutic target (Alquézar et al., 2015; Fan et al., 2017; Wang et al., 2017). In this experiment, β-elemene can reduce cell proliferation by inhibiting the cell cycle, and Cyclin D3 may become one of the targets for β-elemene to reverse the resistance to 5-Fu in p53-deficient colorectal cancer.

In summary, β-elemene can inhibit cell proliferation, increase apoptosis, and enhance 5-Fu sensitivity by inducing pro-death autophagy and inhibiting the progression of the Cyclin D3-dependent cell cycle in p53-deficient colorectal cancer. Pro-death autophagy and cell cycle arrest may be new targets for β-elemene to reverse resistance. β-elemene in combination with 5-Fu will hopefully provide a strategy for the treatment of CRC patients with p53 deletion. However, there are still some limitations in the research. Such as, it is unclear whether there is a correlation between β-elemene-induced pro-death autophagy and Cyclin D3-dependent cell cycle arrest, and there are no clinical data to support our conclusions. In the future, we will make further investigations for this limitations.

## Conclusion

β-elemene can enhance the sensitivity of 5-Fu in p53 wild-type colorectal cancer cells HCT116p53^+/+^. β-elemene can induce pro-death autophagy, affect the expression of cell cycle-related proteins, and cause Cyclin D3-dependent cycle arrest in p53-deficient colorectal cancer cells HCT116p53^–/–^. At the same time, β-elemene can reverse resistance of HCT116p53^–/–^ cells to 5-Fu by inducing pro-death autophagy and Cyclin D3-dependent cycle arrest.

## Data Availability Statement

All datasets generated for this study are included in the article/supplementary material.

## Ethics Statement

The animal study was reviewed and approved by Animal Ethical and Welfare Committee of ZCMU Zhejiang Chinese Medical University.

## Author Contributions

XS, TX, and QW designed the research. RZ, YX, and MZ performed autophagy expriments and cell viability assay. JF, SL, and TD provided technical supports. PC, BZ, WW, RZ, and BC performed animal experiments. XuH, LC, and XC made western blotting analysis. TP, LY, TJ, YL, and GL contributed materials and data analysis. XiH, WZ, YS, QL, QZ, and LZ collected data. RZ wrote manuscript with contributions from the other authors.

## Conflict of Interest

The authors declare that the research was conducted in the absence of any commercial or financial relationships that could be construed as a potential conflict of interest.

## Footnotes

1 http://www.genecards.org
